# Motion as a phenotype: the use of live-cell imaging and machine visual screening to characterize transcription-dependent chromosome dynamics

**DOI:** 10.1186/1471-2121-7-19

**Published:** 2006-04-24

**Authors:** David A Drubin, Arman M Garakani, Pamela A Silver

**Affiliations:** 1 Department of Systems Biology, Harvard Medical School and Department of Cancer Biology, The Dana-Farber Cancer Institute, Boston, MA, USA; 2 Reify Corporation, Cambridge, MA, USA

## Abstract

**Background:**

Gene transcriptional activity is well correlated with intra-nuclear position, especially relative to the nuclear periphery, which is a region classically associated with gene silencing. Recently however, actively transcribed genes have also been found localized to the nuclear periphery in the yeast *Saccharomyces cerevisiae*. When genes are activated, they become associated with the nuclear pore complex (NPC) at the nuclear envelope. Furthermore, chromosomes are not static structures, but exhibit constrained diffusion in real-time, live-cell studies of particular loci. The relationship of chromosome motion with transcriptional activation and active-gene recruitment to the nuclear periphery has not yet been investigated.

**Results:**

We have generated a yeast strain that enables us to observe the motion of the galactose-inducible *GAL *gene locus relative to the nuclear periphery in real-time under transcriptionally active and repressed conditions. Using segmented geometric particle tracking, we show that the repressed *GAL *locus undergoes constrained diffusive movement, and that transcriptional induction with galactose is associated with an enrichment in cells with *GAL *loci that are both associated with the nuclear periphery and much more constrained in their movement. Furthermore, we report that the mRNA export factor Sac3 is involved in this galactose-induced enrichment of GAL loci at the nuclear periphery. In parallel, using a novel machine visual screening technique, we find that the motion of constrained *GAL *loci correlates with the motion of the cognate nuclei in galactose-induced cells.

**Conclusion:**

Transcriptional activation of the *GAL *genes is associated with their tethering and motion constraint at the nuclear periphery. We describe a model of gene recruitment to the nuclear periphery involving gene diffusion and the mRNA export factor Sac3 that can be used as a framework for further experimentation. In addition, we applied to the analysis of chromosome motion a machine visual screening approach that used unbiased visual data rather than segmented geometric data. This novel analytical approach will allow for high-throughput study of processes that can be monitored via alterations in chromosome motion and connectivity with the nuclear periphery.

## Background

The separation of transcription and mRNA processing in the nucleus from cytoplasmic translation affords the eukaryotic cell an added level of regulatory complexity for gene expression. Additionally, the nucleus provides an architectural framework in which the chromosomes are non-randomly organized into chromosome territories, although the position of chromosomes within the nucleus varies between cell types and among populations of cells (for review, see ref. [1]). This variation in chromosome positioning is thought to reflect certain properties of the chromosome, such as size and transcriptional activity [2,3].

The relationship of transcriptional activity with the intra-nuclear positioning of genes has been well documented, particularly relative to the periphery of the nucleus (for reviews, see ref. [4-6]). Localization at the nuclear periphery is traditionally a hallmark of gene silencing. The cystic fibrosis transmembrane regulator (*CFTR*) gene, when silent, is associated with the nuclear periphery, but is more interior when expressed [7]. In the budding yeast *Saccharomyces cerevisiae*, silenced telomeres are localized to the nuclear periphery via the nuclear pore complex (NPC) [8-11], and repression of the silent mating type loci is associated with NPC components and the nuclear periphery [8,9,12].

However, recent studies have suggested that the association with the nuclear periphery is not exclusively for gene silencing. One such study in *S. cerevisiae *demonstrated the activation of a reporter gene by NPC components and nuclear transport machinery via a boundary activity that isolates the gene from silent chromatin [13]. We have since demonstrated through a yeast genome-wide localization analysis that components of the NPC and transport machinery are associated with a subset of actively transcribed genes, as well as the expected silenced genes [14]. Furthermore, we have shown inducible genes involved in galactose metabolism, specifically the *GAL1*, *7*, and *10 *genes (*GAL *locus), shift from the nuclear interior to the NPC upon growth in galactose [14]. We also observed recruitment of genes to the NPC from thirteen different chromosomes that are activated in response to the yeast pheromone alpha factor [15]. Recruitment of the *INO1 *gene to the nuclear periphery upon transcriptional induction [16], and the association of several NPC components with transcriptional activation [17] both provide further evidence that the nuclear periphery is a complex region of transcriptional regulation.

Although there is a defined shift in position of certain activated genes from the nuclear interior to the NPC, the mechanism and functional significance of this phenomenon remain unclear. Chromosomes exhibit a constrained diffusive motion [18-20], which may be a means of bringing genes into association with the nuclear periphery. Transcription itself, as indicated by the RNA dependency for association of alpha factor-induced genes with the NPC [15], may be involved. Furthermore, specific connecting factors may include components of the mRNA export machinery that also exhibit associations with the transcriptional machinery.

In the study presented here, we report that *GAL *locus motion becomes tightly constrained at the nuclear periphery upon transcriptional induction. From this and the observation of transient "sampling" of the nuclear periphery by the *GAL *locus, we propose a model of active-gene recruitment to the nuclear periphery. Furthermore, we present the use of real-time chromosome motion in live cells coupled with machine visual screening as a reporter system for gene localization at the nuclear periphery, and propose its application to other studies of this type.

## Results

### Visualizing GAL locus position and movement in living cells

**Figure 1:**
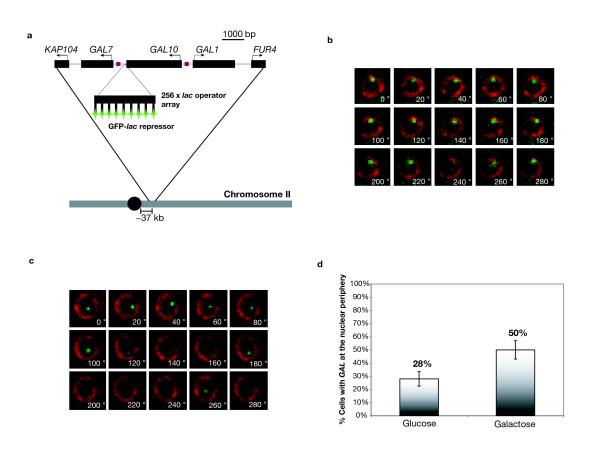
**Live-cell imaging of *GAL *locus intra-nuclear position**. (**a**) A 256-copy *lacO *array (an ~14 kb insert, not drawn to scale) was inserted within the intergenic region between the *GAL7 *and *GAL10 *genes. This array reports the position of the *GAL *locus region relative to the nuclear periphery with the co-expression of the GFP-*lacI *and nucleoporins Nup49 and Nup60 fused to DsRed variant, tdimer2 [23, 24]. (**b**) The image montage is of one nucleus from a field of view of a single z-plane image sequence. Frames represent 20 s samples from a 5 min sequence. *GAL *locus position was scored at the nuclear periphery if the GFP spot mostly overlapped with the red nuclear periphery over the course of an image sequence. (**c**) *GAL *locus position was scored not at the periphery if there was no prolonged signal overlap. (**d**) The percentage of cells that exhibit *GAL *locus association with the nuclear periphery is enriched in galactose-treated populations versus those grown in glucose. Percentages represent four separate experiments with at least 65 cells scored for each treatment group. Error bars represent the standard deviation across experiments.

To study the chromosome dynamics associated with transcription, we tagged the *GAL *locus in a haploid strain of *Saccharomyces cerevisiae *by integrating an array containing 256 *lac *operator *(lacO) *sites into the intergenic region between the *GAL7 *and *GAL10 *genes, and co-expressing a green fluorescent protein-*lac *repressor fusion (GFP-*lacI*); (Figure 1a) [18-22]. The site of integration preserves the promoter elements of *GAL7 *and the 3' elements of *GAL10*, such that the strain exhibits normal regulation of the *GAL *system (data not shown). In addition to marking the *GAL *locus, we also fused two nuclear pore proteins, Nup49 and Nup60, with a yeast codon-optimized DsRed variant, tdimer2 [23,24] in order to mark the nuclear periphery [18].

To determine the ability of our live-cell fluorescence system to correctly report the position of the *GAL *locus, we grew cells in YP-raffinose and split the culture into YP-galactose (induction) and YP-glucose (repression) media for 3-hours. Cultures were then collected and placed on 2% agarose pads on glass slides for imaging. Image sequences (captured every 5 s, for 5 min) were generated from cells in which the *GAL *locus spot was visible and used to score whether the *GAL *locus was at the periphery for the entire time of the image sequence or not (Figure 1b, c). This analysis was repeated for four separate galactose and glucose treatments, and we observed a similar trend as previously reported for *GAL *position [14]; about 50% of cells had the *GAL *locus at the periphery upon transcriptional induction, and only 28% in repressed conditions (Figure 1d).

**Figure 2:**
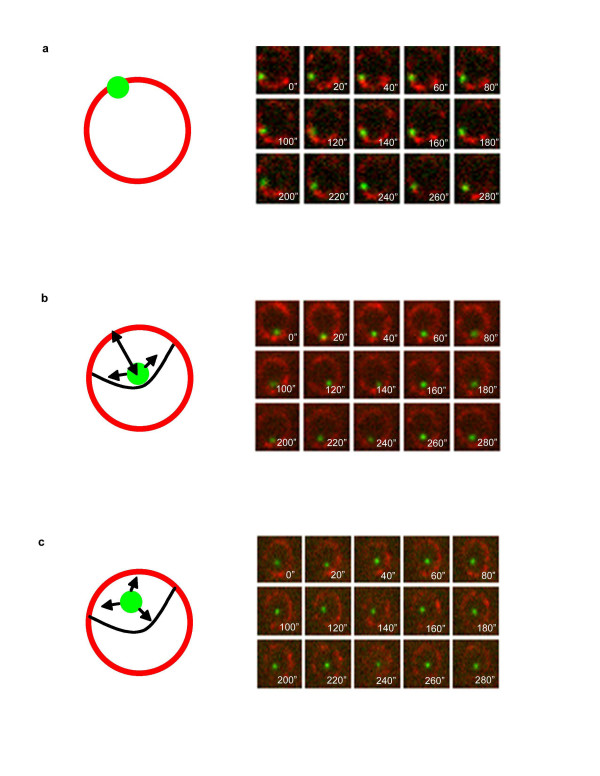
**Three general types of *GAL *locus motion are observed in yeast populations**. (**a-c**) The image montages represent 20 s samples of an at least 5 min image series, where auto-focus was used for the green channel. (**a**) The *GAL *locus is restricted to the nuclear periphery, such that the *GAL *signal visually overlaps with the NPC signal. (**b**) The *GAL *locus is not restricted to the nuclear periphery, but exhibits transient associations. (**c**) The *GAL *locus exhibits no association with the nuclear periphery during the time course of image-capture.

It should be noted that in both transcriptionally induced and repressed cultures we observed the same types of *GAL *locus motion behaviors, but in different proportions. In total, we observed three major types of motion behavior exhibited in cell populations in both glucose and galactose conditions (Figure 2, a-c): loci that are restricted to the nuclear periphery (Figure 2a), transiently associated with the nuclear periphery (Figure 2b), or never associated with the periphery (Figure 2c).

Using manual visual scoring of *GAL *loci at the nuclear periphery in live-cell image sequences, we tested whether a key mRNA export factor, Sac3, was involved in the localization of active genes to the nuclear periphery. We constructed a knockout of *SAC3 *in the *lacO*-tagged *GAL *locus haploid strain. The lack of Sac3 prevented the normal galactose-dependent increase in the per cent of cells with the *GAL *locus at the nuclear periphery (a 22 +/- 6% enrichment in *SAC3(+) *cells, but only a 4 +/- 1% enrichment in *sac3 *mutant cells), supporting a role for this protein in linking active-genes to the NPC.

### *GAL *locus dynamics in transcriptionally induced and repressed conditions

**Figure 3:**
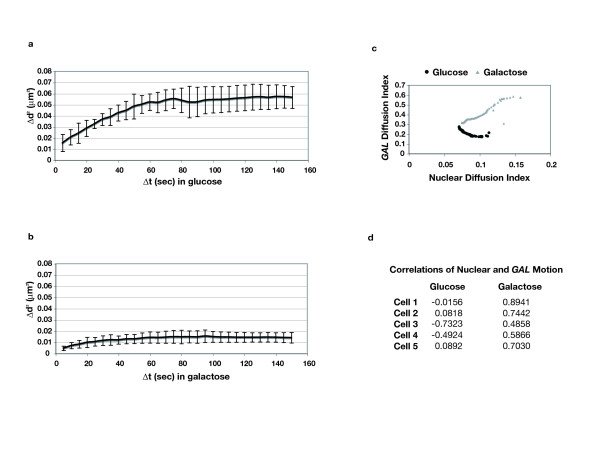
***GAL *locus motion analysis**. (**a, b**) Mean square displacement (MSD) analysis was performed as described [18-20]. The displacement in μm of the *GAL *locus was estimated as the change in distance (*Δd*) over a period of time (*Δt*) in s. The square of the displacement (*Δd*^2^) was calculated as a function of time interval such that *Δd*^2^= {d(*t*)-d(*t*+*Δt*)}^2^, for *Δt *that ranged between 5 to 150 s. The graphs are the average of all *Δd*^2 ^for each *Δt *from five single-nucleus image sequences of at least 5 min in length versus *Δt*. Error bars represent the standard deviation of *Δd*^2 ^for each *Δt *across the five experiments. (**a**) MSD analysis for *GAL *loci not scored at the nuclear periphery in glucose conditions. (**b**) MSD analysis for *GAL *loci scored at the nuclear periphery in galactose conditions. (**c**) The graphs represent examples of the plotted correlation between the nuclear and *GAL *diffusion indices (from *Visible Discovery *software analysis) over all time points of an image sequence for cells grown in galactose (with *GAL *at the nuclear periphery), () or glucose (with *GAL *not at the nuclear periphery); (•). Each point represents the diffusion indices (see text) of the nucleus and *GAL *locus at a specific time *t*. (**d**) The listed values are the Pearson correlations of nuclear and *GAL *diffusion indices for five independent nuclei of cells grown in glucose and galactose conditions (same five cells in each condition as analyzed by MSD analysis in (a) and (b)).

To characterize differences in *GAL *locus motion relative to sub-nuclear location and associated transcriptional activity, we captured single-nucleus image sequences with auto-focus for the *GAL *locus, similar to prior studies [18] of chromosome dynamics. We first compared our data with that previously established for general yeast chromosome motion [18,19], and performed a mean squared displacement analysis (MSD) to determine differences in spatial constraints of diffusion [18,19]. An extension of the open source software ImageJ [25], called SpotTracker [26], was used to align the nuclear periphery signal for each frame to compensate for nuclear motion and subsequently calculate the nuclear center and position of the *GAL *locus [26]. In order for SpotTracker to accurately calculate the nuclear center, parameter optimization was required for the software to effectively find the nuclear periphery. From these data, the distance, d, between nuclear center and *GAL *locus was determined for each frame. For free diffusion, the plot of the square of the average change in d (Δd^2^) versus particular time intervals (Δt) would yield a straight line with a positive slope. Under repressed conditions (in glucose), where *GAL *loci are largely not at the nuclear periphery, we see by MSD calculation that *GAL *locus motion is constrained as it yields a curve demonstrating an increasing slope followed by a plateau (Figure 3a) as previously reported for other chromosomal regions [18,19]. The plateau seen here is at ~0.05 μm^2^, similar to that reported for the centromeres of yeast chromosomes III and IV [18,19]. This represents a confinement radius estimated at ≤ 0.3μm [18,19]. This is consistent with the chromosomal position of the *GAL *locus and *lacO *tag, which are centromere proximal at approximately 37 kb from the centromere of chromosome II (see Figure 1). Not unexpectedly, we find motion of the *GAL *locus much more constrained when associated with the nuclear periphery (Figure 3b), which is enriched for upon galactose induction. The corresponding MSD curve is very similar to that reported for telomeres [18], which are known to be highly associated with the NPC and nuclear periphery [8-10,27,28].

### Machine visual screening of GAL locus motion

To further elucidate the mechanisms of active-gene recruitment to the nuclear periphery, we sought an easier way to study gene localization. Current assays for gene localization at the NPC involve chromatin immunoprecipitation (ChIP), fluorescence in situ hybridization (FISH), or manual analysis of live-cell imaging, all of which are time consuming and/or technically demanding. Here we examine the use of real-time chromosome motion in live cells, coupled with machine visual screening as a reporter system for gene localization at the nuclear periphery. Unlike the previously described MSD analysis employed with SpotTracker, machine visual screening should not involve any pre-analysis parameter optimization or geometric segmentation of the visual data, resulting in easier and more accurate analyses.

To demonstrate the application of machine visual screening to the study of chromosome dynamics, we took the same image series analyzed by traditional geometric methods above and applied the machine visual screening software, *Visible Discovery *[29]. This software generates an aggregate change index (ACI), which is a measure of movement; in this case how spatial organization of fluorescent intensity shifts over time for each frame of the green and red channels. ACI generation does not require parameter optimization or machine-visual segmentation of the *GAL *locus or the nuclear periphery; all intensity values over the period of the experiment are used (see Methods for a mathematical description of ACI). The calculation of ACI for a given frame involves a comparison with all of the other frames in an image series, so that a frame with a large overall change relative to the rest of the image series will have a larger ACI than other frames.

To compare the motion of the nucleus and *GAL *locus described by ACI, a diffusion index was generated for each time point (in each channel) t; the diffusion index is the root mean square of ACI, for the ACI's of all frames up to and including time t. In this fashion, we calculate the way ACI changes over time (see Methods). The diffusion indices of the green channel are then compared via a Pearson correlation to the red channel. If the *GAL *locus were physically connected to the structural framework of the nucleus, such as the nuclear periphery, or both undergoing similar motion patterns, we would find a positive correlation between the two. On the other hand, lack of correlation between the motion pattern of the *GAL *locus and the nuclear periphery would strongly suggest the two are not physically connected. We found that indeed, when the *GAL *locus was scored at the nuclear periphery for cells grown in galactose (the same cells used in the MSD analysis), the correlation between the two channels shows a consistent positive correlation (above ~0.5). In contrast, the correlation of the channels from glucose-grown cells for which the *GAL *locus was not scored at the periphery ranged from around 0 to strongly negative (Figure 3c and 3d).

## Discussion

The transcriptional regulation of genes has been linked to their sub-nuclear positioning, particularly relative to the periphery of the nucleus. The nuclear periphery has been traditionally characterized as a zone of repression. However, recent work from our laboratory and others showing the presence of active genes [14-17] has established that the regulatory milieu at the nuclear periphery is more intricate than first thought.

There are several prevailing ideas as to how and why activated genes relocate to the nuclear periphery. Work by Menon *et al *[17] suggests that NPC components themselves are involved in transcriptional activation, and that genes are repositioned in the nucleus in order to be brought into a concentrated region of transcription factors for activation. Our laboratory has recently shown the opposite, that the interaction of the NPC component Mlp1 with genes induced by the yeast mating pheromone alpha factor is dependent upon RNA, suggesting that gene association with the nuclear periphery occurs after transcription has begun [15]. Work by Brickner and Walter [16] detailing recruitment of the *INO1 *locus to the nuclear periphery upon induction, demonstrated that artificial recruitment of the *INO1 *gene to the nuclear membrane altered the regulation of the gene, but did not directly result in its activation. To better clarify the mechanism behind active-gene recruitment to the NPC in yeast, we assessed the transcription-associated chromosome dynamics in live-cells, specifically of the inducible *GAL *gene locus.

We confirmed that *lacO*/GFP-*lacI *tagging of the *GAL *locus was representative of *GAL *localization. Previously, via FISH and NPC-immunofluorescence (FISH/IF), we demonstrated that the *GAL *locus was at the nuclear periphery with a slightly greater enrichment than observed here. However, the image sequence element of this work may result in differences, as several *GAL *loci scored at the nuclear periphery in the FISH/IF technique likely represent transient associations at the periphery. Furthermore, the percentages reported here are similar to those observed in the fixed-image, *lacO*/GFP-*lacI *reporting of the *INO1 *intra-nuclear position in response to transcriptional induction [16].

The *lacO*-tagging technique has been previously used to track the motion of several different chromosome regions in yeast [18,19]. In these prior studies, the motion of chromosomes is defined as constrained diffusion, and our data is consistent with this classification. One study, using a similar 2-dimensional tracking, showed that telomeric regions exhibited the greatest motion constraint throughout interphase, while centromeres had an intermediate constraint, and ARS regions had the least constraint [18]. Furthermore, S-phase and the process of replication resulted in the specific increase in constraint of ARS-region motion [18].

Here we have shown a similar "constraining" phenomenon for the *GAL *locus that is dependent upon transcription-activating conditions, and associated with the specific position of the locus at the nuclear periphery. The *GAL *genes are located near (~37 kb) the centromere of yeast chromosome II, and, as expected, exhibit a similar degree of motion constraint when not restricted to the NPC as that reported for the centromeres of yeast chromosomes III and IV [18,19]. When found positioned at the nuclear rim, *GAL *locus motion was highly constrained, comparable to previous observations for telomeric regions [18] and suggesting a physical anchorage to the NPC [14]. Such restriction in movement is in agreement with data showing peripheral localization correlates with increased motion constraint in human cells [30]. This notion is further supported by analysis with the *Visible Discovery *software [29], in which the motion of the *GAL *locus is positively correlated with the movement of the nucleus when induced and at the periphery.

Interestingly, when not restricted to the nuclear rim, the *GAL *locus often exhibited transient overlaps with the nuclear periphery. This is significant as these transient interactions may bring specific factors (nuclear pore and gene-associated) into proximity. The result of a successful interaction would be *GAL *locus constraint at the nuclear membrane, which is what we observe upon *GAL *induction. That we still see a fraction of cells with *GAL *loci not at the periphery in galactose-induced populations may reflect the stochasticity of gene expression in a population of cells (for review see ref. [31]), coupled with a certain probability of gene-NPC interaction. The probability of interaction would be a function of both the chance of a transient interaction of the *GAL *gene with the NPC resulting from a chromosome's constrained random walk and the on-rate of the interacting factors.

### A model for dynamic genomic compartmentalization involving transcription and mRNA export

If our model suggests there is an increased affinity of the gene for the nuclear periphery upon transcription-activating conditions, what might be responsible for this increased affinity? Previous work from our lab has suggested that RNA is a mediating factor of active-gene and NPC component connectivity [15]. This suggests that a number of mRNA-binding proteins and export factors, which have an affinity for the NPC and associated components, could bridge the interaction between a transcribing gene and the NPC. In these studies, we showed that at least one key factor involved in transcriptional enrichment of loci at the nuclear periphery is the mRNA export protein Sac3. Given the vast array of interactors documented for Sac3 [32-35], there are a number of possibilities for how Sac3 may link activated genes to the nuclear periphery. Sac3 is localized at the NPC [32-34,36], and interacts with transport factors [32,34] and a component of the transcription-activating SAGA complex [37], Sus1 [35]. Interestingly, the localization of Sus1 at the nuclear periphery is disrupted in the absence of Sac3 [35], perhaps indicating a lack of SAGA-dependent genes, such as the *GAL *genes [38-42], at the nuclear rim.

**Figure 4:**
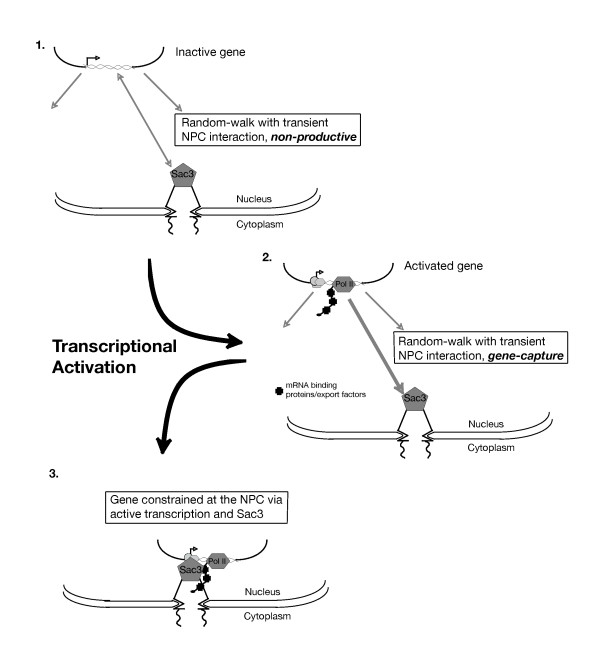
**A model for active-gene recruitment to the NPC**. When repressed and inactive, an inducible gene exhibits nucleoplasmic diffusion with occasional transient sampling of the nuclear periphery and NPC. Upon transcriptional induction, the transcription machinery is recruited to the gene promoter and transcription is initiated. Appropriate RNA-binding proteins are recruited and deposited upon the nascent mRNA. The activated gene locus now contains many factors with an affinity for NPC-associated Sac3. Once the active locus samples the nuclear periphery after nascent transcription has begun, the gene is captured and constrained at the NPC via Sac3.

Based on these data, we propose a working model for active-gene recruitment to the NPC (Figure 4). As transcription occurs, the appropriate mRNA binding proteins are recruited and deposited on the nascent transcript. It is at this point we propose the cognate gene region has acquired an increased affinity towards the NPC, via Sac3. The active-gene region, which now hosts many Sac3-interacting proteins, is now primed for capture at the NPC.

### Chromosome dynamics as a potential phenotypic screen

Upon characterizing the motion of the *GAL *loci by the previously employed segmented geometric particle tracking methods, we desired to harness the differences in motion observed between NPC localized *GAL *and sub-nuclear *GAL *to aid in the further phenotypic characterization of tethering of active genes to the NPC. To this end, we tested the *Visible Discovery *machine visual screening analysis software, which is more automated than geometric particle-tracking methods. We demonstrated that, when corralled at the nuclear periphery, the motion of the *GAL *locus was correlated with that of the nucleus, as inferred by automated measurements of movement indices. This not only reinforces the model that genes become physically associated with the NPC at the nuclear envelope, but also demonstrates that movement is a phenotype that can be easily queried.

The geometric particle tracking methods (such as the MSD analysis) require various estimations and assumptions about the data for analysis and currently requires extensive tweaking and manipulation of the data for the software to "find" the nuclear periphery and spot center. The *Visible Discovery *analysis platform frees us from these restraints and tedious preparations, and is much more amenable to adaptation for high-throughput analysis. In this case, a visual field of live cells with a tagged *GAL *locus and nuclear periphery could be monitored over a time course and each nucleus analyzed. (To eliminate the need for auto-focus on one cell at a time, a z-stack of the field could be captured and projected as a 2-dimensional frame for each time point.) A percentage of cells in which the *GAL *locus motion is correlated with nuclear motion could then be quickly calculated. This motion correlation could, conceptually, also be used for screens of gene associations with different nuclear compartments. For our purposes, to make sure a gene showing correlated motion with the nucleus was actually associated with the nuclear periphery, a simple automated check of signal localization could be made.

An experimental screen would involve the perturbation of the system via mutants or chemical treatments in which this percentage of cells would be assessed for change. In such a way, the molecular details of the mechanism linking active genes with the NPC could be elucidated. Furthermore, the use of motion as an easily screened phenotype could be employed for the studies of many other systems. As we have defined a means of easily monitoring the connectivity of a gene with components of the nuclear periphery, any process involving a gene-nuclear rim association, such as yeast telomere positioning, could be monitored in this fashion.

## Conclusion

This work demonstrates the transcription-associated dynamics of the inducible *GAL *locus in yeast populations, and a method for machine-screening chromosome localization. Observations of *GAL *locus movement in populations of transcriptionally induced and repressed conditions lead us to a proposed model for recruitment of an activated gene to the NPC: recruitment results from the random sampling of the nuclear periphery by a gene locus undergoing diffusion that is "captured" upon activation by a mechanism dependent upon Sac3 and nascent mRNA (Figure 4). The machine visual screening analysis, coupled with a high throughput study design, will allow for the easier screening of mutants or chemical treatments on active gene recruitment to the NPC in order to further dissect the molecular mechanism of the process. Furthermore, such a motion phenotype-based screen could be applied to the study of other functions involving alterations in chromosome motion and/or nuclear sub-compartmentalization.

## Methods

### Yeast strains, plasmids, and growth conditions

To integrate the *lacO *array at the *GAL *locus between *GAL7 *and *GAL10*, a 1 kb region containing the *GAL7/10 *intergenic region was first PCR amplified from a chromosome preparation of haploid strain PSY2156 (MAT**a**, *ade2-1*, *trp1-1*, *can1-100*, *leu2-3*, *112*, *his3-11*, *15*, *ura3*) [14,15,43] using the following primer sequences: 5'-GTGAGCTCCTATTGGTAACCATACAG-3' and 5'-GTGGTACCGAGGCAAAGCAATTTAAG-3', such that the ends of the PCR product contained SacI and KpnI sites. The PCR fragment was cut and ligated into the pAFS59 vector containing the *lacO *array [19,21,22], resulting in plasmid pPS3112. pPS3112 was linearized for integration using NcoI. The gene encoding GFP-*lacI *was integrated at *HIS3 *by cutting plasmid pAFS135 with NheI [19,21,22]. Fluorescently tagged nucleoporins, Nup49 and Nup60, were generated via C-terminal fusions of the native genes with a yeast-optimized DsRed variant, tdimer2, using PCR-amplified cassettes [23,24]. The final strain was PSY3364 (MAT**a**, *ade2-1*, *trp1-1*, *can1-100*, *NUP49*-tdimer2,*NUP60-*tdimer2, *lacO*x256, GFP-*lacI*). For knockouts of *SAC3 *in PSY3364 (creating PSY3368), a PCR-amplified knockout cassette was used, encoding the nourseothricin resistance marker [44]. All integrations were verified by PCR. All growth is at 30°C unless noted otherwise.

For the comparison of galactose induction and glucose repression, cells were first grown from a streaked plate overnight in a 10 mL culture in YP media containing 2% raffinose. As the yeast strains used were *ade2*-, 20 μg/mL of adenine (Sigma-Aldrich, Inc, St. Louis, MO) was added. Cultures of mid-log phase density were separated in half the next day, both halves spun down, and one half re-suspended in 10 mL of YP media containing 2% galactose and the other half re-suspended in 10 mL of YP media containing 2% glucose. Re-suspended cultures were grown for 3-hours.

### Sample preparation and image capture

Samples were prepared on plain microslides that were coated with 2% YP-agarose His- medium with 2% glucose or galactose. For slide coating, ~6 μL of a heated 2% agarose solution was applied to a slide and a coverslip placed on top. The agarose was allowed to harden for at least 40 minutes and the coverslip was slid off so as to preserve a thin coating on the slide. Cells were harvested by centrifugation, washed once with water, and resuspended in 100 μL of minimal media with 2% glucose or 2% galactose. About 4 μL of cell suspension was placed on the agarose coated slide and a 22 × 22 mm, no. 1½, coverslip applied. Coverslips were sealed with Valap (1:1:1 petroleum jelly, lanolin, and paraffin). Cells were imaged using a Nikon TE2000U inverted microscope with PerkinElmer ultraview spinning disk confocal and a 100 × oil objective. For collecting a sequence of a single z-plane field of cells, cells were imaged at 23°C for 5 min with capture every 5 s. Exposure for the green channel (488 nm excitation) was 300 ms, and the red channel (568 nm excitation) was 700 ms using a binning of 2, such that 1 pixel = 0.13 μm. Sequences of single cells for tracking were captured in a similar fashion for at least 5 min, except auto-focus on the *GAL *locus was used in Metamorph 6.3r2 (Molecular Devices, Chicago, IL).

### Data analysis

For visually scoring *GAL *loci position in an image series, loci visible for most of the 5 min time course were counted at the periphery if locus-overlap with the NPC signal appeared continuous, others were counted as not at the periphery. Cells with two loci were not scored. About ten different fields of view were captured as an image sequence for each treatment or growth condition, at least in triplicate. Cells with more than two visible loci, and those with deformed nuclear envelopes, were not counted.

We used both an established as well as a novel method to determine the extent to which the motion pattern of the *GAL *locus was constrained or freely diffusive. Identical image sequences of individual nuclei (five for each condition, glucose and galactose) were used for all analysis.

#### SpotTracker

Single particle tracking methods using mean square displacement (MSD) have been previously established for quantifying movement characteristics. SpotTracker is a plug-in for the software ImageJ [25]. SpotTracker algorithms and parameter sets have been previously detailed [26]. SpotTracker effectively assigns x,y coordinates of the nuclear center and GAL locus spot for each frame. For each image sequence, calculated *GAL *locus and nuclear center coordinates were input into Microsoft Excel to calculate the distance between them (*d*) and the Δ*d*^2 ^as a function of the time interval (Δ*t*) such that Δ*d*^2^={d(*t*)-d(*t*+Δ*t*)}^2^. A set of user parameters for intensity thresholds, fitting parameters, and confidence thresholds, dependent upon the image sequence, were optimally set for this analysis. These values and settings were set empirically based upon the raw image data being analyzed.

#### Machine visual screening

*Visible Discovery *(Reify Corporation, Cambridge, MA) [29] is a proprietary machine visual screening software platform that we used here to calculate two parameters, the diffusion index (Ds), and the Aggregate Change Index (ACI). These parameters are defined for an ordered series of *n *frames defined by an intensity function *I*_*x,y,t*_, where *x *and *y *are integers defining the coordinates of a given pixel in a particular frame, and *t *is an integer corresponding to the time of capture. Frames are captured at equally spaced intervals. The equations below are based on the more general equations given in [45].

First, a similarity function *SM *is calculated for all pairs of frames taken at times *i *and *j *(*i *≠ *j*):



Where *N*= *wh*, and each double summation is calculated over the width *w *and height *h *of the frame; i.e., ∑x=0w∑y=0h
 MathType@MTEF@5@5@+=feaafiart1ev1aaatCvAUfKttLearuWrP9MDH5MBPbIqV92AaeXatLxBI9gBaebbnrfifHhDYfgasaacH8akY=wiFfYdH8Gipec8Eeeu0xXdbba9frFj0=OqFfea0dXdd9vqai=hGuQ8kuc9pgc9s8qqaq=dirpe0xb9q8qiLsFr0=vr0=vr0dc8meaabaqaciaacaGaaeqabaqabeGadaaakeaadaaeWbqaamaaqahabaaaleaacqWG5bqEcqGH9aqpcqaIWaamaeaacqWGObaAa0GaeyyeIuoaaSqaaiabdIha4jabg2da9iabicdaWaqaaiabdEha3bqdcqGHris5aaaa@3ADC@, and is calculated for all pairs of *i *and *j *where *i *≠ *j*. The similarity function is commutative, such that *SM*_*i,j *_= *SM*_*j,i*_. Furthermore, *SM*_*i,j *_= 1 if i==j.

A normalized *SM*_*i,j *_function, called *P*_*i,j *_is then calculated for each *SM*_*i,j *_of an image series.

Pi,j=SMi,j∑k=0nSMi,k     (2)
 MathType@MTEF@5@5@+=feaafiart1ev1aaatCvAUfKttLearuWrP9MDH5MBPbIqV92AaeXatLxBI9gBaebbnrfifHhDYfgasaacH8akY=wiFfYdH8Gipec8Eeeu0xXdbba9frFj0=OqFfea0dXdd9vqai=hGuQ8kuc9pgc9s8qqaq=dirpe0xb9q8qiLsFr0=vr0=vr0dc8meaabaqaciaacaGaaeqabaqabeGadaaakeaacqWGqbaudaWgaaWcbaGaemyAaKMaeiilaWIaemOAaOgabeaakiabg2da9maalaaabaGaem4uamLaemyta00aaSbaaSqaaiabdMgaPjabcYcaSiabdQgaQbqabaaakeaadaaeWbqaaiabdofatjabd2eannaaBaaaleaacqWGPbqAcqGGSaalcqWGRbWAaeqaaaqaaiabdUgaRjabg2da9iabicdaWaqaaiabd6gaUbqdcqGHris5aaaakiaaxMaacaWLjaWaaeWaaeaacqaIYaGmaiaawIcacaGLPaaaaaa@49A9@

The Aggregate Change Index for a given frame *i *is calculated as follows:

ACIi=∑j=0nPi,jlog⁡2(Pi,j)/log⁡2(n)     (3)
 MathType@MTEF@5@5@+=feaafiart1ev1aaatCvAUfKttLearuWrP9MDH5MBPbIqV92AaeXatLxBI9gBaebbnrfifHhDYfgasaacH8akY=wiFfYdH8Gipec8Eeeu0xXdbba9frFj0=OqFfea0dXdd9vqai=hGuQ8kuc9pgc9s8qqaq=dirpe0xb9q8qiLsFr0=vr0=vr0dc8meaabaqaciaacaGaaeqabaqabeGadaaakeaacqWGbbqqcqWGdbWqcqWGjbqsdaWgaaWcbaGaemyAaKgabeaakiabg2da9maaqahabaGaemiuaa1aaSbaaSqaaiabdMgaPjabcYcaSiabdQgaQbqabaGccyGGSbaBcqGGVbWBcqGGNbWzdaWgaaWcbaGaeGOmaidabeaakmaabmaabaGaemiuaa1aaSbaaSqaaiabdMgaPjabcYcaSiabdQgaQbqabaaakiaawIcacaGLPaaacqGGVaWlcyGGSbaBcqGGVbWBcqGGNbWzdaWgaaWcbaGaeGOmaidabeaakmaabmaabaGaemOBa4gacaGLOaGaayzkaaaaleaacqWGQbGAcqGH9aqpcqaIWaamaeaacqWGUbGBa0GaeyyeIuoakiaaxMaacaWLjaWaaeWaaeaacqaIZaWmaiaawIcacaGLPaaaaaa@5712@

The Diffusion Index *Ds *is calculated based on ACI as:



In such a way, *Visible Discovery *computes a diffusion index from an ACI for the independent image sequences (green channel and red channel) of both the *GAL *locus and nuclear periphery. No pre-processing or parameter tuning was applied. Software execution time for a single movie was less than a 100 ms on a 1 Ghz Macintosh G4 running OS X.

ACI is a self-similarity temporal index (see [46] for characterization of Brownian motion as a self-similar process) and is an estimate of the temporal autocorrelation function. A diffusion index at any time point t is the root mean square of ACI values from and including the first time point up to t. Without compensation for drifts, ACI assumes that instantaneous displacements are within a small envelope of the autocorrelation function.

Random diffusion or transport can be characterized either from correlation of velocities, or correlation of positions [47]. MSD analysis calculates the average of displacements over fixed interval times, i.e. a velocity correlation function. ACI is a position correlation function, i.e. variations of correlation of a process calculated spatially over time. ACI analysis does not attempt to model the underlying process. Rather, ACI computes a trend of how the underlying process changes over time. Computing ACI, requires comparing every image in the sequence to every other image. Image sequences were 2 dimensional arrays, or regions within, of pixel values. We used normalized cross-correlation to measure the similarity function between two images.

## Authors' contributions

DAD carried out all the biological aspects of the study, performed the geometric MSD analysis, participated in the experimental design and conceptualization of the project, and drafted the manuscript. AMG supplied software expertise and design in performing all the machine visual screening analysis and its description in the manuscript. PAS participated in the design and coordination of the study and helped in the process of developing the manuscript. All authors read and approved the final manuscript.
